# Failed Awake Flexible Intubation Followed by Early Surgical Tracheostomy in Advanced Laryngeal Cancer: A Case Report

**DOI:** 10.1155/cria/5110841

**Published:** 2026-08-26

**Authors:** Bosung Kim, Haesung Lee, Ji-Hyoung Park

**Affiliations:** ^1^ Department of Anesthesiology and Pain Medicine, Wonju College of Medicine, Yonsei University, Wonju 26426, South Korea, unifesp.br

**Keywords:** airway management, airway obstruction, intubation, squamous cell carcinoma of larynx, tracheostomy

## Abstract

Primary laryngeal cancers causing critical airway stenosis pose significant challenges to airway management. Although flexible bronchoscope guided awake intubation is generally recommended for anticipated difficult airways, the presence of a friable and obstructive mass may render this technique ineffective. We report the case of a 69‐year‐old male who presented with dyspnea and stridor due to a T4a transglottic carcinoma involving the vocal cords and anterior commissure. Despite a carefully planned awake flexible bronchoscopic intubation, advancement of the endotracheal tube was impossible due to the tumor’s mass effect. Consequently, the airway management strategy was promptly changed to an emergency surgical tracheostomy under local anesthesia, which was successfully performed by an otolaryngologist on standby without perioperative complications. This case highlights the limitations of noninvasive techniques in the presence of severe anatomical distortion and emphasizes the importance of multidisciplinary preparation for immediate front‐of‐neck access in high‐risk laryngeal cancer patients.

## 1. Introduction

Airway management is a fundamental competency for anesthesiologists, and the ability to secure the airway safely remains a critical determinant of perioperative patient safety. Patients with advanced laryngeal tumors represent a particularly challenging subgroup because progressive airway narrowing may render conventional airway management strategies ineffective despite preservation of spontaneous ventilation [1]. Such events pose immediate life‐threatening risks and may rapidly progress to severe hypoxic brain injury or death, underscoring the continued clinical relevance of timely airway rescue strategies.

Primary laryngeal and glottic cancers are relatively uncommon but represent a unique challenge with respect to airway management. Globally, they account for approximately 1% of all cancers [2]. Although these tumors are often characterized as slow growing, local invasion into structures such as the vocal ligament, intrinsic laryngeal musculature, or anterior commissure may result in vocal cord fixation, progressive airway narrowing, and potential obstruction [3]. These anatomical alterations can introduce significant unpredictability during airway management, particularly at induction of anesthesia.

According to the 2022 American Society of Anesthesiologists (ASA) difficult airway guidelines, awake intubation is recommended when difficult tracheal intubation is anticipated, particularly in patients who may not tolerate apnea or in whom emergency invasive airway access may be difficult. However, these guidelines provide limited discussion regarding patients with severe upper airway obstruction caused by laryngeal tumors, in whom awake intubation may fail despite preserved spontaneous ventilation [4]. In such patients, early conversion to a surgical airway before the development of a true Cannot Intubate, Cannot Oxygenate (CICO) event may represent a safer airway management strategy [4].

Both the ASA 2022 and the Difficult Airway Society (DAS) 2025 guidelines emphasize limiting repeated airway attempts and prompt transition to an alternative airway strategy when the initial airway plan fails [4, 5]. However, neither guideline provides specific recommendations for patients with advanced laryngeal tumors who maintain spontaneous ventilation despite failed awake intubation. In such patients, early surgical tracheostomy before the development of a true CICO event may represent a safer and more controlled airway management strategy. Herein, we report a case of failed awake flexible intubation followed by early surgical tracheostomy in a patient with advanced laryngeal cancer causing critical glottic obstruction.

## 2. Case Presentation

A 69‐year‐old male, 155.5 cm in height and 56.4 kg in weight with BMI 23.3, presented to the emergency department via ambulance with complaints of dyspnea, oxygen desaturation, and audible stridor. His medical history was significant for hypertension managed with medication and a 20‐pack‐year smoking history. He had no known drug allergies or previous adverse medication reactions.

**FIGURE 1:**
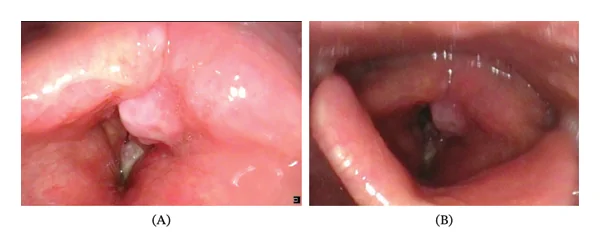
Laryngoscopy performed three months before emergency presentation. (A) Vocal cords in the closed position showing significant airway narrowing caused by a fungating mass in the left glottic region. (B) Despite maximal abduction, the glottic airway remains markedly restricted due to the obstructing lesion.

Six months prior, the patient visited a local clinic for new‐onset voice changes and was referred to an otorhinolaryngology clinic due to concern for possible laryngeal malignancy. He presented to the clinic three months later, at which time laryngoscopic examination revealed a fungating mass involving the left glottic region (Figure 1). Although immediate treatment was recommended, the patient declined intervention at that time due to personal social circumstances.

**FIGURE 2:**
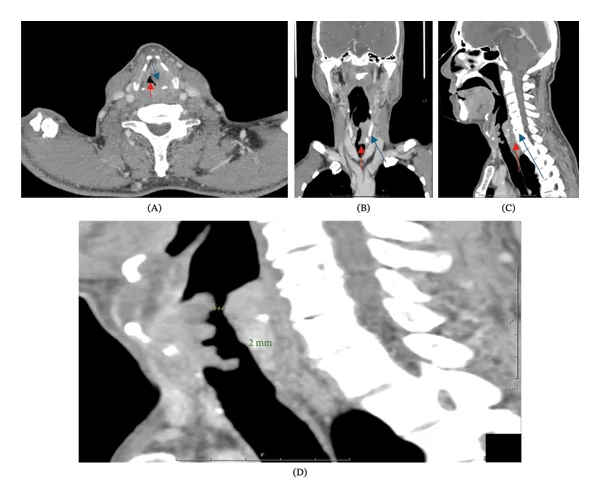
Preoperative contrast‐enhanced CT images of the neck. (A) Axial, (B) coronal, and (C) sagittal views demonstrating a transglottic laryngeal tumor. Red arrows indicate the markedly narrowed residual airway lumen, whereas blue arrows indicate the transglottic tumor. (D) Magnified sagittal CT image demonstrating the narrowest residual airway lumen, measuring approximately 2 mm.

All laboratory parameters were within normal limits, whereas arterial blood gas analysis revealed an elevated PCO_2_ of 47 mmHg. A contrast‐enhanced CT of the neck demonstrated an enhancing mass at the left true vocal cord with supraglottic and infraglottic extension, as well as paraglottic spread suggestive of thyroid cartilage invasion (Figure 2). The minimum residual airway lumen measured approximately 2 mm at the level of the vocal cords, indicating critical airway narrowing and a potentially difficult airway (Figure 2D). An outside contrast‐enhanced neck CT demonstrated an enhancing transglottic mass involving the left true vocal cord with supra‐ and infraglottic extension, paraglottic extension, and suspected thyroid cartilage invasion without radiologically enlarged cervical lymph nodes, suggesting cT4aN0 disease. Further staging evaluation at our institution included contrast‐enhanced CT of the chest and abdomen, which showed no evidence of distant metastasis, and contrast‐enhanced MRI of the larynx. MRI demonstrated a 1.5 cm heterogeneous left level IIa cervical lymph node suspicious for nodal metastasis, and the tumor was, therefore, clinically staged as cT4aN1M0 according to the AJCC 8th edition [6].

Transoral microsurgery under general anesthesia was planned for tissue biopsy and relief of airway obstruction. As this patient was anticipated to have a difficult airway, preprocedural airway planning was performed according to the ASA difficult airway guidelines. Difficult tracheal intubation was suspected based on preoperative airway evaluation including video laryngoscopy, and difficulty in ventilation using a facemask or supraglottic airway device was also anticipated. Laryngoscopy performed 3 months prior to the operation showed a slit like opening which led to the decision of trying endotracheal intubation to secure the airway instead of immediate cricothyroidotomy or surgical access of the airway. Therefore, awake airway management was planned, with high‐flow nasal oxygen for adequate preoxygenation, and preparation of a flexible bronchoscope and 6.0‐mm nasal endotracheal tubes for intubation. An otolaryngologist was also notified and remained available to perform emergent tracheostomy in the event of failed awake intubation or progression to a true CICO situation. Based on the preoperative CT findings, emergency cricothyrotomy was considered anatomically inappropriate because the tumor was located immediately posterior to the cricothyroid membrane. Performing a cricothyrotomy would have required incision through the tumor, raising concerns regarding bleeding and potential tumor seeding.

The patient entered the operating room with a blood pressure of 187/81 mmHg, heart rate of 87 beats per minute, and peripheral oxygen saturation of 99%. Mallampati score class II, mouth opening 3 finger width, thyromental distance 3 finger, and neck extension were in difficulty due to orthopnea. Preprocedural airway ultrasonography was not performed. Instead, the airway was evaluated using preoperative contrast‐enhanced CT, and the cricothyroid membrane was identified by palpation. However, cricothyrotomy was not considered an appropriate rescue strategy because the tumor was located immediately posterior to the cricothyroid membrane, raising concerns regarding tumor violation, bleeding, and potential tumor seeding.

Intravenous glycopyrrolate (0.1 mg) was administered as premedication to reduce airway secretions. High‐flow nasal oxygen (FiO_2_ 1.0, flow rate 40 L/min) was applied in the sitting position for preoxygenation. Midazolam (3 mg IV) was administered for anxiolysis, followed by topical airway anesthesia using twenty sprays of 10% lidocaine applied to the oropharynx, tonsillar pillars, and base of the tongue. Intravenous propofol (20 mg) was then administered to facilitate awake flexible intubation while preserving spontaneous ventilation.

Awake flexible intubation was attempted using a flexible bronchoscope loaded with a 6.0‐mm right‐angle nasal endotracheal tube, which was selected based on the residual glottic opening observed on preoperative laryngoscopy performed 3 months earlier. However, the tube could not be advanced beyond the vocal cords because of obstruction by the fungating tumor, which had progressed considerably over the preceding 3 months. A second awake flexible intubation attempt using a 5.5‐mm endotracheal tube performed by another experienced anesthesiologist was also unsuccessful. A microlaryngeal tube, Tritube, and a Ventrain device were unavailable at our institution. Because of the patient’s progressive airway obstruction and failure of two awake intubation attempts, the procedure could not be deferred. After confirming that spontaneous ventilation and adequate oxygenation were maintained following withdrawal of the flexible bronchoscope and endotracheal tube, the anesthesiology and otolaryngology teams jointly decided to proceed with surgical tracheostomy under local anesthesia. Additional sedation was achieved with intravenous propofol (40 mg). High‐flow nasal oxygen was maintained throughout the airway management process. During preparation for surgical tracheostomy, gentle facemask ventilation was attempted; however, effective ventilation remained limited because of the severe glottic obstruction. Despite this, oxygen saturation remained at 100% throughout the approximately 3 min period required to establish the surgical airway.

**FIGURE 3:**
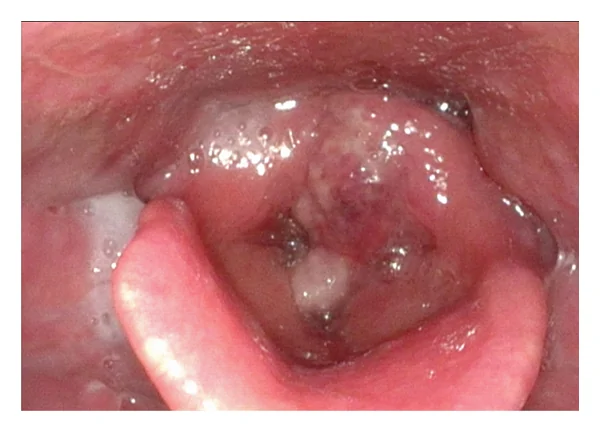
Intraoperative laryngoscopic view. Compared with three months prior, the enlarging mass further compromises the glottic airway opening.

Following successful tracheostomy, general anesthesia was induced with 50 mg of rocuronium, and surgery commenced. Arterial blood gas analysis was unremarkable. Intraoperative laryngoscopic examination revealed a large fungating mass involving the left arytenoid cartilage, extending over the left true vocal cord, the anterior commissure, and into the subglottic region (Figure 3). Histopathological examination of the biopsy specimen confirmed squamous cell carcinoma of the larynx. Partial tumor debulking was also performed to relieve the airway obstruction.

Neuromuscular blockade was reversed with sugammadex, and the patient was transferred to the postanesthesia care unit after full recovery of consciousness with spontaneous breathing through the tracheostomy tube.

Ten days later, definitive surgery consisting of total laryngectomy, total thyroidectomy, and lymph node dissection was performed, and a tracheoesophageal voice prosthesis (Provox®, Atos Medical) was inserted. The 9‐h procedure proceeded without intraoperative complications, and the patient was transferred to the surgical intensive care unit for postoperative management.

The patient returned to the operating room 3 hours later due to postoperative bleeding, during which a minor bleeding vessel was identified and cauterized. Hemoglobin levels showed only a minimal decrease from 10.3 to 9.8 g/dL, and the patient was transferred back to the surgical intensive care unit for further postoperative management.

Extubation was successfully performed two days later, and the patient was subsequently transferred to the general ward. After gradual dietary progression, the patient was discharged 33 days after admission, with plans for adjuvant radiotherapy. The chronological clinical course is summarized in Table 1.

**TABLE 1:** Timeline of major clinical events from initial symptom presentation to discharge.

Time	Event
Day (‐179)	First visit to local physician with voice change
Day (‐87)	Visit to ENT outpatient clinic; flexible laryngoscopy revealed a fungating mass on the left glottic area; treatment deferred due to patient’s personal circumstances
Day 0 (03:04)	Arrival at emergency department via ambulance with dyspnea; oxygen administered
Day 0 (06:33)	Hospital admission
Day 0 (12:25)	Transfer to operating room for airway intervention
Day 0 (12:30)	High‐flow nasal oxygen applied (SpO_2_ 100%)
Day 0 (12:35)	Awake intubation attempted; ENT surgeon on standby for emergency tracheostomy
Day 0 (12:40)	Intubation failed; tracheostomy initiated (SpO_2_ 100%)
Day 0 (12:45)	Tracheostomy completed; mechanical ventilation started (FiO_2_ 0.40, tidal volume 450 mL, respiratory rate 16/min, PEEP 5 cm H_2_O)
Day 0 (14:00)	Extubation performed; transfer to general ward
Day 10	Definitive surgery (total laryngectomy, thyroidectomy, lymph node dissection, and voice prosthesis insertion)
Day 12	Extubation performed at surgical intensive care unit
Day 33	Discharge with plan for adjuvant radiotherapy

*Note:* The chronological sequence summarizes the patient’s clinical course, including early evaluation for dysphonia, subsequent identification of a glottic mass, acute respiratory deterioration requiring emergent airway management on the day of admission (Day 0), definitive oncologic surgery on hospital Day 10, and discharge on hospital Day 33 with plans for adjuvant radiotherapy.

## 3. Discussion

This case highlights the complexity of airway management in patients with laryngeal malignancy causing critical airway narrowing. Although awake flexible endoscopic intubation is routinely recommended for anticipated difficult airways in current ASA guidelines [4], the presence of an obstructing glottic mass can significantly limit the feasibility and safety of the technique. In this patient, preoperative neck CT and laryngoscopic evaluation clearly demonstrated a fungating lesion with near‐complete glottic obstruction, allowing the anesthesiology team to anticipate a difficult airway and prepare accordingly. Despite adequate preoxygenation and appropriate use of flexible endoscopic equipment, advancement of the endotracheal tube was unsuccessful due to tumor‐related narrowing, necessitating prompt surgical tracheostomy. Although emergency front‐of‐neck access is recommended once a true CICO situation develops, our patient maintained spontaneous ventilation and adequate oxygenation throughout the failed awake intubation attempt. Therefore, this case is more appropriately characterized as failed awake flexible intubation followed by early surgical tracheostomy, rather than a true CICO event. This case highlights the importance of timely conversion to a surgical airway before the onset of hypoxemia in selected patients with advanced laryngeal tumors. The decision to attempt awake flexible intubation rather than primary surgical tracheostomy was based on the residual slit‐like glottic opening observed on preoperative laryngoscopy, together with preservation of spontaneous ventilation and adequate oxygenation. Under these circumstances, awake flexible intubation was considered a reasonable initial strategy while ensuring immediate availability of surgical tracheostomy if tube passage proved unsuccessful. However, primary surgical tracheostomy may represent the preferred initial airway strategy in patients with more advanced airway obstruction or when preoperative imaging and endoscopic findings indicate that successful endotracheal tube passage is unlikely. In the present case, interval tumor progression rendered endotracheal tube advancement impossible despite preserved spontaneous ventilation, ultimately necessitating early surgical tracheostomy.

The DAS 2025 and ASA 2022 difficult airway guidelines both emphasize the importance of early recognition and rapid progression to alternative strategies in the event of failed airway attempts [4, 5]. The DAS algorithm for unanticipated difficult intubation limits tracheal intubation attempts to three, followed by supraglottic airway attempts, and mandates declaration of a CICO situation before performing emergency front‐of‐neck access [5]. The ASA guideline similarly prioritizes awake intubation for anticipated difficulty but underscores continuous readiness to proceed to emergency invasive airway access [4]. Importantly, both guidelines highlight minimizing repeated attempts that may contribute to airway trauma or loss of oxygen reserve. However, neither the ASA nor the DAS guideline provides specific recommendations for selecting between awake flexible intubation and primary surgical tracheostomy in patients with advanced laryngeal tumors causing critical airway obstruction. Airway management in these patients should, therefore, be individualized based on preoperative imaging, endoscopic findings, and multidisciplinary discussion.

Airway obstruction due to laryngeal tumors introduces additional challenges that are not fully addressed by standard difficult airway algorithms. Mass lesions can obscure flexible endoscopic visualization, distort anatomical landmarks, or collapse dynamically during manipulation [7, 8]. Furthermore, insertion of a bronchoscope or endotracheal tube may paradoxically precipitate complete obstruction. For these reasons, several experts advocate considering awake tracheostomy as the primary approach in cases with severe structural obstruction [9]. In our patient, awake flexible intubation was initially selected because spontaneous ventilation and oxygenation were preserved, and preoperative imaging suggested that a residual glottic lumen remained, making endotracheal tube passage appear potentially feasible. Nevertheless, this case highlights that primary awake tracheostomy should be considered earlier in selected patients with advanced laryngeal tumors when severe glottic obstruction makes successful tube passage unlikely, even if oxygenation is preserved.

This case also illustrates the coexistence of difficult airway and potentially difficult front‐of‐neck access [10]. Conditions such as upper tracheal stenosis, morbid obesity with limited neck extension, traumatic neck swelling, or deep neck infection may complicate surgical approaches. Although the patient in this case did not exhibit these features, clinicians must remain vigilant for scenarios where both routes may be challenging, prompting early multidisciplinary coordination with otolaryngology.

There were notable strengths in the management of this case, including accurate prediction of airway difficulty based on imaging, comprehensive preparation of equipment, preprocedural patient counseling, and early involvement of an otolaryngologist. Additionally, the decision to transition promptly to surgical tracheostomy following failed awake intubation prevented unnecessary airway trauma and avoided critical desaturation. Limitations of this case include the inability to secure the airway using flexible endoscopic techniques despite thorough preprocedural planning. Nevertheless, this outcome is more indicative of the inherent unpredictability and dynamic nature of tumor‐related airway obstruction than of inadequate procedural preparation or execution.

In summary, this case highlights the importance of individualized airway planning in patients with laryngeal tumors, particularly in situations where guideline‐recommended airway strategies may not be feasible due to anatomical distortion. Early consideration of surgical airway access, coordinated multidisciplinary involvement, and strict adherence to established principles of difficult airway management are critical to ensuring safe and effective care in these high‐risk patients.

## Author Contributions

Conceptualization: Bosung Kim and Ji‐Hyoung Park.

Writing–original draft: Bosung Kim and Haesung Lee.

Writing–review and editing: Bosung Kim and Ji‐Hyoung Park.

Supervision: Ji‐Hyoung Park.

## Funding

The authors received no financial support for this publication.

## Consent

Written informed consent was obtained from the patient for publication of this case report and accompanying images.

## Conflicts of Interest

The authors declare no conflicts of interest.

## Data Availability

Data sharing is not applicable to this article as no datasets were generated or analyzed.
